# Atomic rearrangement of a sputtered MoS_2_ film from amorphous to a 2D layered structure by electron beam irradiation

**DOI:** 10.1038/s41598-017-04222-6

**Published:** 2017-06-20

**Authors:** Bong Ho Kim, Hyun Ho Gu, Young Joon Yoon

**Affiliations:** Nano-Convergence Materials Center, Korea Institute of Ceramic Engineering and Technology, 101, Soho-ro, Jinju-si, Gyeongsangnam-do 52851 Republic of Korea

## Abstract

We synthesised a crystalline MoS_2_ film from as-sputtered amorphous film by applying an electron beam irradiation (EBI) process. A collimated electron beam (60 mm dia.) with an energy of 1 kV was irradiated for only 1 min to achieve crystallisation without an additional heating process. After the EBI process, we observed a two-dimensional layered structure of MoS_2_ about 4 nm thick and with a hexagonal atomic arrangement on the surface. A stoichiometric MoS_2_ film was confirmed to grow well on SiO_2_/Si substrates and include partial oxidation of Mo. In our experimental configuration, EBI on an atomically thin MoS_2_ layer stimulated the transformation from a thermodynamically unstable amorphous structure to a stable crystalline nature with a nanometer grain size. We employed a Monte Carlo simulation to calculate the penetration depth of electrons into the MoS_2_ film and investigated the atomic rearrangement of the amorphous MoS_2_ structure.

## Introduction

Two-dimensional (2D) transition metal dichalcogenides (TMDCs), which show a semiconducting band structure unlike graphene with a zero bandgap^1^, have recently attracted a high level of interest because of their remarkable electronic and optical performance^2, 3^. In particular, atomically thin molybdenum disulphide (MoS_2_) films, which have a thickness-dependent optical bandgap ranging from 1.3 eV (bulk, indirect) to 1.8 eV (monolayer, direct)^4^, a quasiparticle bandgap of 2.84 eV^5^ and spin-orbit splitting^6^, have been investigated as one of the most promising TMDCs for next-generation electronic and optoelectronic applications, such as transistors^7–9^, photodetectors^10^, and solar cells^11^. However, a direct growth technique needs to be developed that is compatible with conventional silicon technology and can be integrated with flexible substrates at sufficiently low temperature to extend the applicability of MoS_2_ films to various electronic devices.

Through similar synthetic methods evolved from graphene research like mechanical exfoliation and transfer, monolayer and few-layer MoS_2_ films can be easily prepared on various substrates^7–9^. Although exfoliation is the simplest technique for demonstrating the features of MoS_2_ films, it is limited to synthesising monolayer or few-layer MoS_2_ films at the flake scale. In addition, controlling the number of layers precisely is difficult, and the fabrication process is susceptible to contamination.

Recently, chemical vapour deposition (CVD) and physical vapour deposition (PVD) methods have been explored to realise the wafer-scale growth of MoS_2_ films for mass production. CVD methods that use MoS_2_^12^, molybdenum(V) chloride (MoCl_5_)^13^, molybdenum trioxide (MoO_3_)^14^, and ammonium thiomolybdate ((NH_4_)_2_MoS_4_)^15^ as precursors have been reported. However, ensuring the uniformity of grown films in a wafer-scale and high-temperature heating process over 650 °C is still a challenge. In PVD methods, similar problems related with scaling up and the operating temperature still exist. A hybrid sputtering process combined with CVD, which first deposits a Mo film and then progresses to sulfurization via a chemical reaction with sulphur vapour, has recently been reported, but it need sufficient heating of the substrate to over 600^16^ or 800 °C^17^. There have been attempts to carry out deposition and sulfurization simultaneously by using a Mo target in hydrogen sulphide (H_2_S) gas^18^ and vaporised sulphur ambient^19^. However, these processes also require heating to a high temperature of up to 700–750 °C. Several researchers have reported a direct sputtering process with a MoS_2_ target at a relatively low substrate temperature of 300–350 °C, but as-deposited MoS_2_ films show poor crystallinity compared to other methods^20–22^. Recent studies on the post-processing of as-deposited MoS_2_ films with thermal annealing^23^ and laser treatment^24^ showed improved crystallinity, but the problems of high temperature and high cost remain.

In this paper, we suggest a simple method of sputtering and post-processing with electron beam irradiation (EBI) to obtain crystalline MoS_2_ films at a low temperature below 100 °C. The amorphous structure of as-deposited MoS_2_ films can be transformed into a crystalline nature after only electron beam irradiation (EBI) with an energy of 1 kV for 1 min. There have been several reports on exploring the structural evolution of materials from amorphous to crystalline as they are being transformed at the local region through the application of a high-energy electron beam of several hundred kilovolts to several megavolts in transmission electron microscopy (TEM)^25–28^. Interestingly, our EBI process makes it possible to stimulate the atomic rearrangement of amorphous MoS_2_ film under the conditions of a relatively low electron energy of 1 kV and short time of 1 min. We think that this EBI technique can be easily applied to scale up the synthetic process of MoS_2_ films considering its compatibility with conventional PVD processes such as sputtering and evaporation.

## Results and Discussion

**Figure 1:**
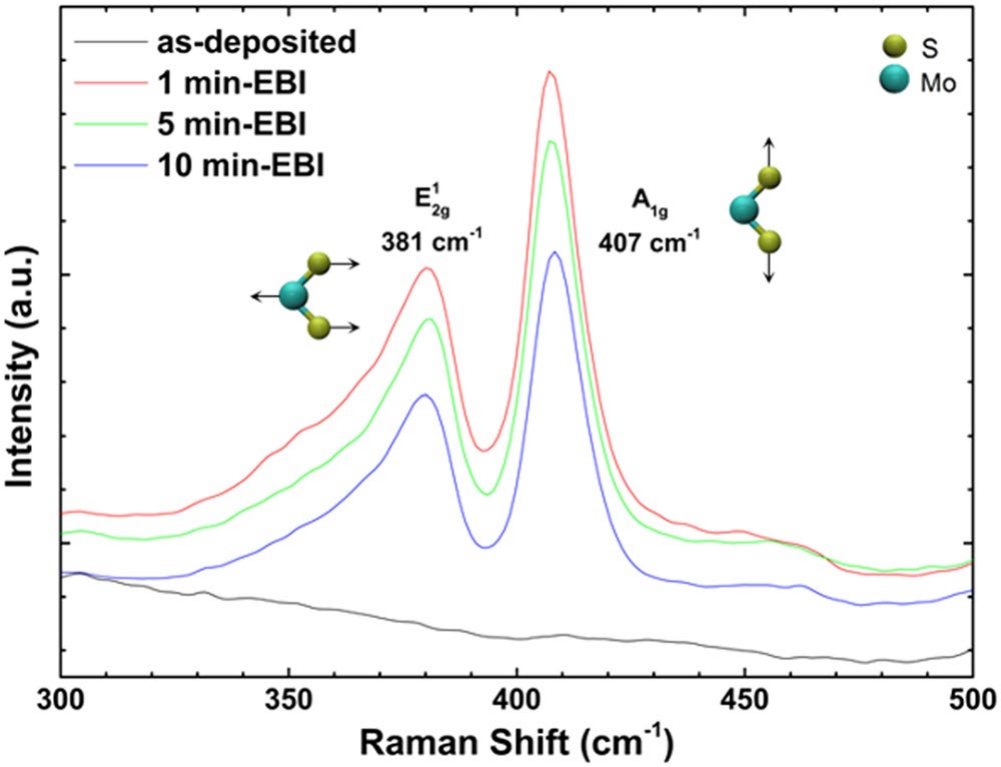
Raman spectra of as-deposited and EBI-treated MoS_2_ films according to irradiation times of 1, 5, and 10 min.

Figure 1 shows the Raman spectra of MoS_2_ films synthesised by using sputtering and the EBI process with different irradiation times. In the as-deposited film grown at room temperature with a working pressure of 5 mTorr, two prominent Raman peaks of MoS_2_, i.e. the in-plane mode (E^1^_2g_) and out-of-plane mode (A_1g_), did not appear because of its amorphous nature. However, after the EBI process with as-deposited sample, the peak intensities of the E^1^_2g_ (~381 cm^−1^) and A_1g_ (~407 cm^−1^) modes increased dramatically without an additional thermal heating process. As the EBI process time was increased from 1 min to 10 min, the peak intensities of the E^1^_2g_ and A_1g_ bands decreased without peak shifts. The highest intensities of the E^1^_2g_ and A_1g_ peaks for the 1 min EBI-treated sample indicated that the amount of Mo–S bonding may decrease with a longer irradiation time. The difference between the Raman shifts of the E^1^_2g_ and A_1g_ peaks (*∆k*) was ~25 cm^−1^, which is typically shown for six atomic layers^5^ or bulk MoS_2_^29^.

For the 1, 5, and 10 min EBI-treated samples, as shown in Table 1, the full width at half maximum (FWHM) values of E^1^_2g_ were 19.3, 17.6, and 19.1 cm^−1^, respectively, and those for A_1g_ were 11.5, 11.6 and 12.1 cm^−1^, respectively. The FWHM of the EBI sample was higher than that of the exfoliated monolayer MoS_2_ (FWHM of E^1^_2g_: 2.8 cm^−1^, FWHM of A_1g_: 4.7 cm^−1^)^30^. However, the crystallinity of our 1 min EBI-treated sample at room temperature was superior to that of sputtered MoS_2_ films (FWHM of E^1^_2g_: ~50 cm^−1^, FWHM of A_1g_: ~14 cm^−1^) with a substrate temperature of 300 °C^20^ and comparable to that of 5.4 nm thick MoS_2_ films (FWHM of E^1^_2g_: ~12 cm^−1^, FWHM of A_1g_: ~14 cm^−1^) grown at 600 °C with a hybrid process of sputtering combined with the CVD method^16^. Especially, instead of the long tail for the E^1^_2g_ peak that is commonly observed in the Raman spectra of a MoS_2_ film from the sputtering process^20–22^, a relatively short tail was observed in our results. Considering the physical meaning of FWHM and intensity values of Raman peaks, which are closely related with the crystal quality for the layered structure of MoS_2_, we can deduce that an irradiation time of over 1 min does not greatly improve the crystallinity of a MoS_2_ film but should decrease the amount of Mo–S bonding because of the loss of sulphur caused by the heating effect^20^ and the phenomenon of Auger decay^31^.

**Table 1:** FWHM of E^1^_2g_ and A_1g_ peaks in Raman spectra (Fig. 1) and average values of E^1^_2g_, A_1g_ peak positions, and *∆k* in Raman map data (Fig. 2) for 1, 5, and 10 min EBI-treated samples.

Sample	FWHM (cm^−1^)		Average (cm^−1^)		
	E^1^_2g_	A_1g_	E^1^_2g_	A_1g_	*∆k*
1 min	19.3	11.5	381.44	406.77	25.33
5 min	17.6	11.6	381.25	406.69	25.44
10 min	19.1	12.1	381.42	406.64	25.22

**Figure 2:**
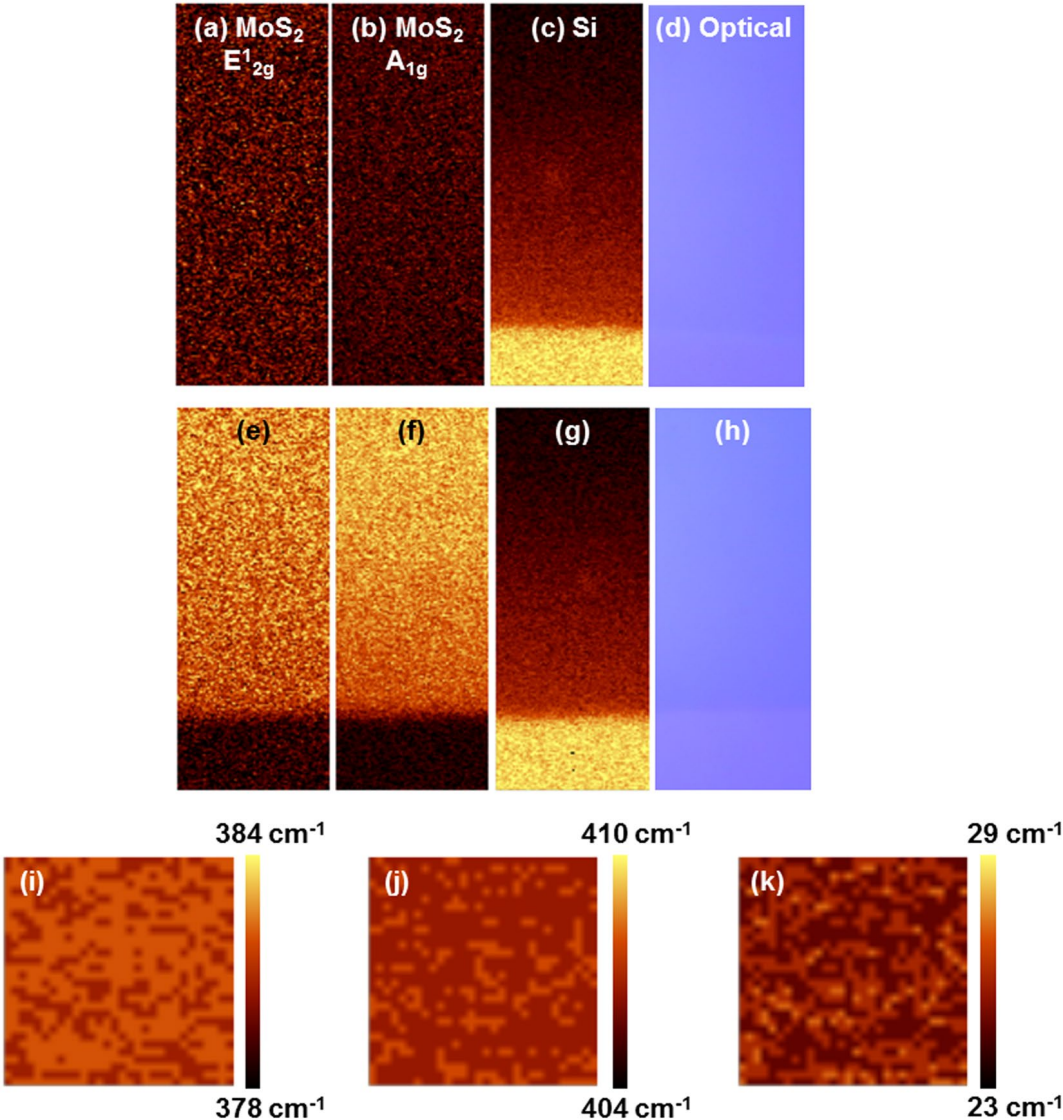
Raman mapping of samples (**a–h**) at the edge and (**i–k**) at the centre of the MoS_2_ films. (**a**–**d**) as-deposited samples and (**e**–**h**) samples treated with EBI for 1 min at the edge of MoS_2_ (20 µm × 50 µm). (**a**,**e**) E^1^_2g_ mode, (**b**,**f**) A_1g_ mode, and (**c**,**g**) Si peaks were scanned at 375–385, 405–411, and 517–523 cm^−1^, respectively. (**d**,**h**) Optical images of scanned area. (**i**–**k**) Raman mapping of the peak position for the 1 min EBI-treated sample at the centre of MoS_2_ (30 µm × 30 µm). (**i**) E^1^_2g_ mode, (**j**) A_1g_ mode, and (**k**) ∆k.

Figure 2(a–h) show Raman mapping data and optical images of as-deposited and 1 min EBI-treated samples over an area of 20 µm × 50 µm for integrated peak intensities within Si (517–523 cm^−1^), E^1^_2g_ (375–385 cm^−1^), and A_1g_ (405~411 cm^−1^) wavenumbers. In the case of the as-deposited sample shown in Fig. 2(a–c), neither the E^1^_2g_ nor A_1g_ peak from the MoS_2_ film was observed because of its amorphous structure, while only the strong intensity from the first-order peak of silicon appeared because of the substrate. Figure 2(e) and (f) show the 1 min EBI-treated sample and confirm that the crystalline MoS_2_ film was uniformly synthesised on the SiO_2_/Si substrate based on the high-intensity maps for the E^1^_2g_ and A_1g_ peaks. Unlike the as-deposited sample, the boundary between the MoS_2_ film and SiO_2_/Si substrate can be clearly observed in the Raman mapping data (Fig. 2(g)), and it is in good agreement with that in the optical image (Fig. 2(h)). Figure 2(i) and (j) show the dispersion of the Raman shift of the E^1^_2g_ and A_1g_ peaks over the area of 30 µm × 30 µm from the centre region of the 1 min EBI-treated sample. The Raman shift of the E^1^_2g_ peak dispersed at 381–382 cm^−1^ with an average value of 381.44 cm^−1^, and the Raman shift of the A_1g_ peak dispersed at 406–408 cm^−1^ with an average value of 406.77 cm^−1^. The average peak difference (*∆k*) was 25.33 cm^−1^, which corresponding to the value of six atomic layers or bulk MoS_2_. Even when the EBI time was increased to 5 and 10 min, *∆k* did not change significantly as summarised in Table 1. Based on this comparison, we estimated that the crystallinity of the EBI-treated samples was improved significantly through the atomic rearrangement of the Mo and S atoms from amorphous to a crystalline-layered structure.

**Figure 3:**
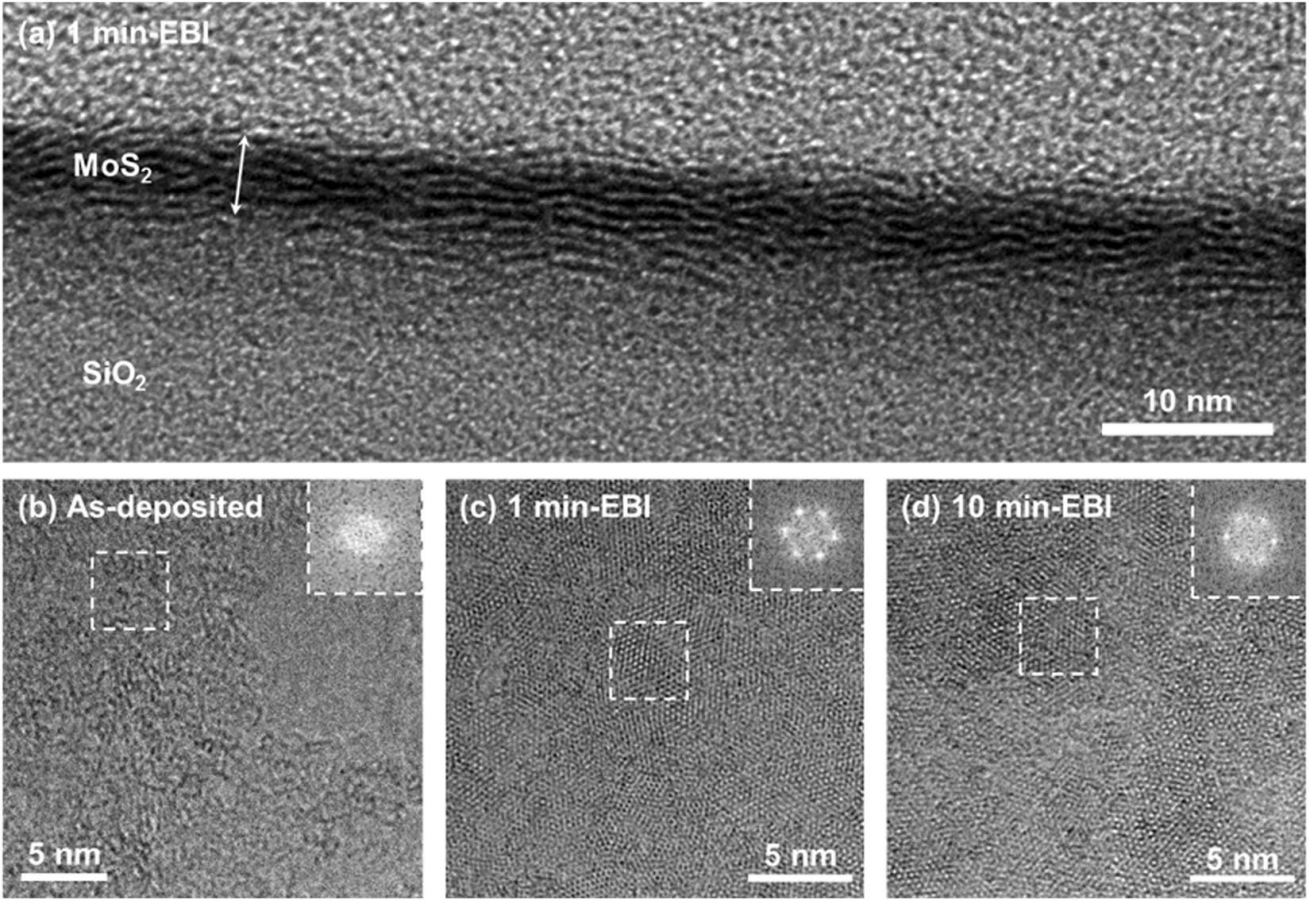
HR-TEM images of EBI-treated MoS_2_ films. (**a**) Cross-sectional HR-TEM image of 1 min EBI-treated MoS_2_ film. Plan-view HR-TEM images of the (**b**) as-deposited sample, (**c**) 1 min EBI-treated sample, and (**d**) 10 min EBI-treated sample. Insets show FFT patterns of the areas marked as dashed squares.

High-resolution TEM analysis was performed in order to confirm the crystalline microstructure of the MoS_2_ films after EBI, as discussed in the results of the Raman analysis. Figure 3(a) shows a cross-sectional TEM image of a synthesised MoS_2_ film on a SiO_2_/Si wafer obtained by room-temperature sputtering with EBI as a post-process for 1 min. Five to seven atomic layers of MoS_2_ formed parallel to the substrate with a thickness of ~4 nm. Even though the size of the MoS_2_ crystal domain was limited to about 5 nm, this is clear evidence of the atomic rearrangement from amorphous to a crystalline structure by the EBI process. Plan-view TEM images of the as-deposited and 1 and 10 min EBI-treated samples are shown in Fig. 3(b)–(d), respectively. Figure 3(b) shows that the amorphous nature was observed over the entire as-deposited sample. However, crystallites with a size of ~5 nm were distributed in an area of 20 nm × 20 nm for the 1 and 10 min EBI-treated samples, as shown in Fig. 3(c) and (d). This indicates a startling transformation of the MoS_2_ film from amorphous to a crystalline structure by the EBI process within 1 min. In the 10 min EBI-treated sample, however, the area of the amorphous region was slightly increased compared to that of the 1 min EBI-treated sample. This may be from the Mo–S bonds breaking owing to the excessive energy transfer with the longer irradiation time. The insets of Fig. 3(b)–(d) show the fast Fourier transform (FFT) patterns from the areas marked as dashed squares. They reveal that the crystal domain of the MoS_2_ film transformed from an amorphous state into a hexagonal lattice structure. The FFT patterns of the as-deposited sample showed typical amorphous characteristics with a wide halo ring. In contrast, sharp diffraction spots in the FFT patterns of the 1 min EBI-treated sample indicated that the *c*-axis of the crystal structure (space group P6_3_/mmc) was perpendicular to the substrate. The FFT pattern in Fig. 3(d) shows that the crystalline ordering for hexagonal symmetry of the 10 min EBI-treated sample was somewhat inferior to that of the 1 min EBI-treated sample.

**Figure 4:**
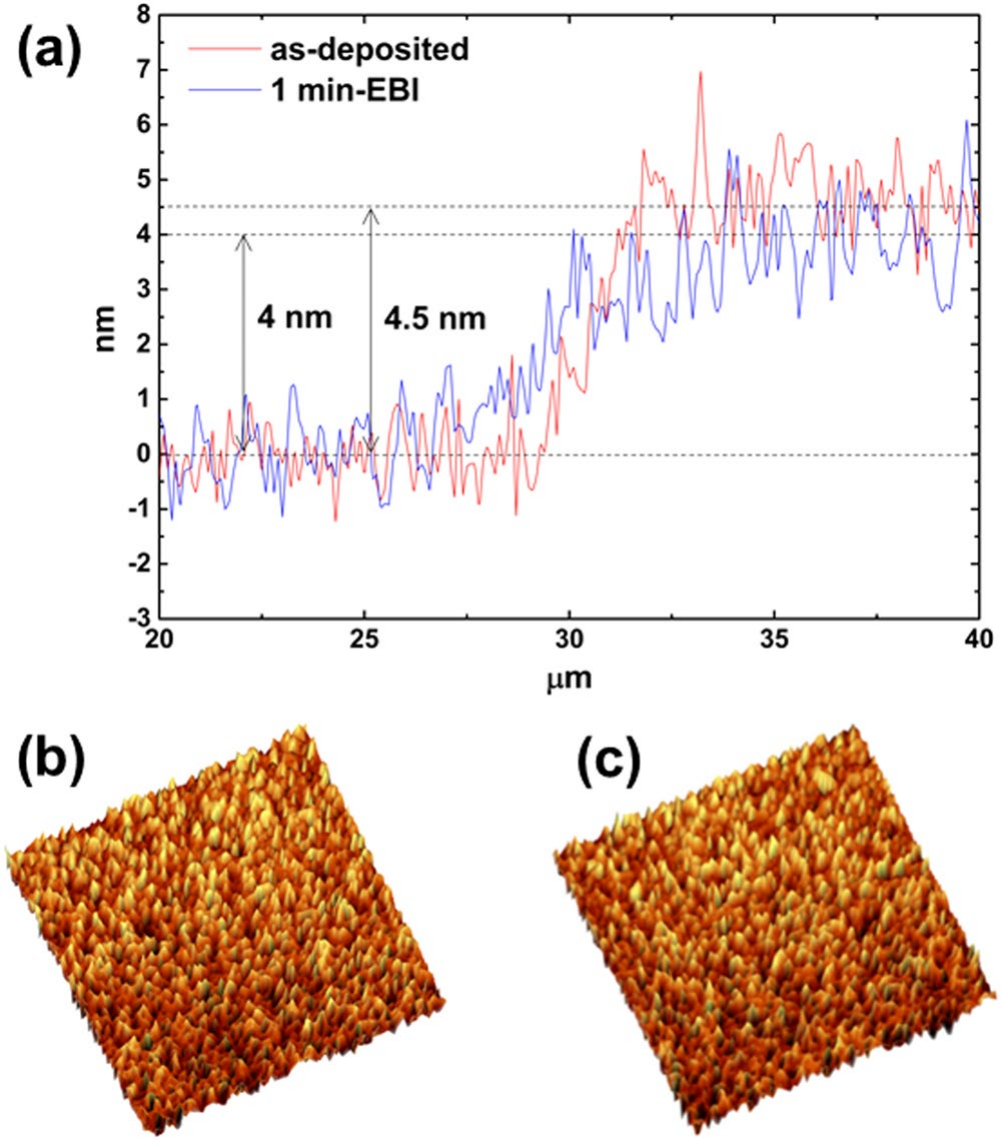
AFM results. (**a**) AFM height profiles of the as-deposited and 1 min EBI-treated sample. AFM images of the (**b**) as-deposited sample and (**c**) 1 min EBI-treated sample in the area of 5 µm × 5 µm.

Figure 4(a) shows the atomic force microscopy (AFM) height profiles at the edges of the as-deposited and 1 min EBI-treated MoS_2_ films. The average thickness of the MoS_2_ film slightly decreased after the EBI process from 4.5 nm to 4.0 nm. Figure 4(b) and (c) show AFM images of the as-deposited and 1 min EBI-treated samples. However, the roughness measured at the centre region of the MoS_2_ films changed significantly with the EBI time. The AFM roughness (*R*_*a*_) values of the SiO_2_ substrate and as-deposited sample were almost the same at 0.524 and 0.523 nm, respectively. The *R*_*a*_ values of the 1, 5, and 10 min EBI-treated samples were 0.696, 0.572 and 0.541 nm, respectively. Because of the atomic rearrangement from amorphous to crystalline MoS_2_, the roughness inevitably increased owing to the formation of crystallites. However, *R*_*a*_ became close to that of the as-deposited sample with a longer EBI time.

**Figure 5:**
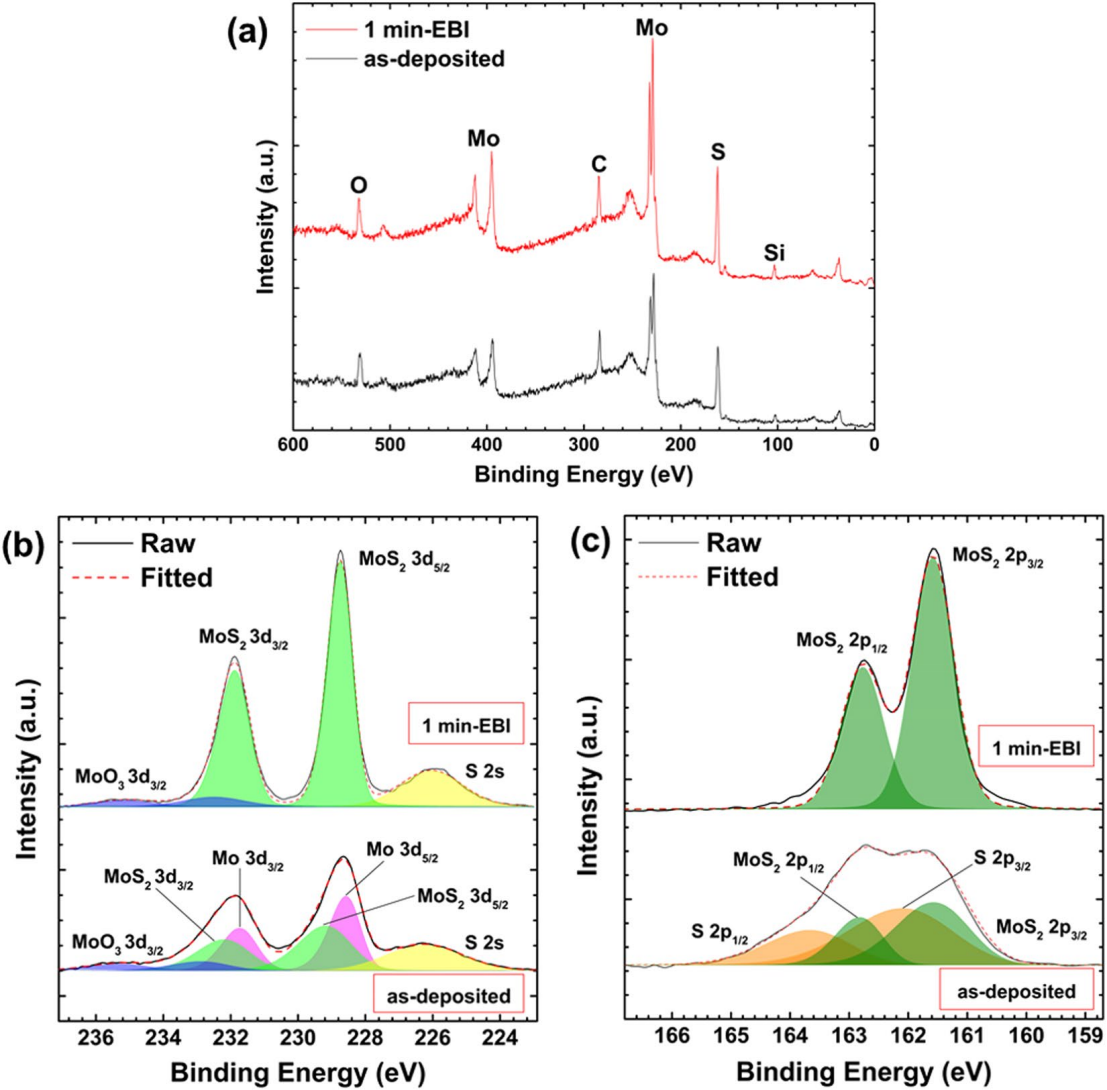
XPS spectra of the (**a**) survey spectrum, (**b**) Mo 3d spectrum, and (**c**) S 2p spectrum from as-deposited and 1 min EBI-treated samples.

We performed X-ray photoelectron spectroscopy (XPS) analysis on the as-deposited and 1 min EBI-treated samples to examine the change in chemical composition after crystallisation with the EBI process. Figure 5(b) and (c) show the high-resolution narrow scan spectra of Mo 3d and S 2p, respectively, including the curve fitting results. The spectrum shift due to sample charging was calibrated by adjusting the carbon 1 s peak to 284.5 eV. In the as-deposited sample, the Mo 3d peak was deconvoluted into three chemical bonding states of Mo–Mo, Mo–S, and Mo–O components. These correspond to the Mo 3d_5/2_ peaks at 228.6, 229.2, and 235.2 eV, respectively. On the other hand, in the case of the 1-min EBI-treated sample, the Mo 3d spectrum consisted of two peaks originating from the states of the Mo–S and Mo–O bonds. These corresponded to the Mo 3d_5/2_ peaks at 228.7 and 235.2 eV, respectively. The chemical bonding state of Mo–Mo was observed to disappear after the EBI process, but the peak intensity from the Mo–S bond increased dramatically. The chemical bonding between Mo and O in the as-deposited sample indicates the formation of MoO_3_, which seems to have originated from the interaction at the interface between the MoS_2_ film and SiO_2_ substrate. This result is similar to previous reports^22, 23^. In this work, the interaction at the interface between the SiO_2_ substrate and MoS_2_ film seems dominant because the intensities of the oxygen and silicon peaks in the XPS survey spectra increased slightly with the 1 min EBI, as shown in Fig. 5(a). The increased intensity indicates a decrease in the MoS_2_ thickness, which would increase the number of electrons escaping from the SiO_2_ substrate and Mo–O bonding at the interface. However, Mo atoms at the interface prefer to bond with S rather than O because Mo–O bonding simply originates from the bond of Mo and O atoms. Therefore, with the 1 min EBI, the ratio of O decreased from 17.7% to 16.4%, while ratio of Si increased from 5.4% to 7.5%.

Similarly, high-resolution XPS spectra of the S 2p peak from as-deposited sample were deconvoluted into S–S and S–Mo bonding that corresponded to the S 2p_3/2_ peaks at 162.1 and 161.6 eV, respectively. In the case of the 1 min EBI-treated sample, only the S–Mo bond remained, showing its 2p_3/2_ peak at 161.6 eV. After the precise deconvolution of the Mo 3d and S 2p spectra, the intensities of each peak were determined to quantify the bound states in MoS_2_ films. Table 2 summarises the peak intensities. In the as-deposited sample, the intensity ratios of the Mo–O, Mo–S and Mo–Mo bonds from the Mo 3d peak were 11.54%, 44.53%, and 43.93%, respectively. Those of the S–S and S–Mo bonds from the S 2p peak were 57.32 and 42.68%, respectively. The atomic ratio of S/Mo was calculated from the Mo–S bonds to be 2.24 in the as-deposited sample. A large portion of the Mo–Mo and S–S bonds was observed to still remain. In the 1 min EBI-treated sample, the intensity ratios of the Mo–O and Mo–S bonds from the Mo 3d peak were 8.63% and 91.37%, respectively. In the case of the S 2p peak, only the S–Mo bonds remained, and the atomic ratio of S/Mo was exactly 2. However, considering the residual Mo–O bonds, we can estimate that the 1 min-EBI sample showed a slightly S-deficient state.

**Table 2:** Area ratio of bonding states after the deconvolution of Mo 3d and S 2p spectrums and the calculated value of the S/Mo ratio from the as-deposited and 1 min EBI-treated samples.

Sample	Mo 3d			S 2p		S/Mo ratio
	Mo–O	Mo–S	Mo–Mo	S–S	S–Mo	
as-deposited	11.54	44.53	43.93	57.23	42.68	2.24
1 min-EBI	8.63	91.37	—	—	100	2.00

The XPS results showed many bonding states in the as-deposited sample, such as Mo–S, Mo–Mo, S–S, and Mo–O. Because of the different sputter yields of Mo and S from the MoS_2_ compound target, the surface composition of the target changed until a steady state was reached when the sputtered flux was S/Mo = 2, which represents the composition of MoS_2_. If both S and Mo have the same sticking coefficient on the SiO_2_/Si substrate, the film would be MoS_2_. However, S has a lower sticking coefficient than Mo, and the film was slightly deficient in sulphur under the normal sputtering conditions. For S to have a sticking coefficient of 1, two S atoms would have to react with a Mo atom as they arrive on the substrate. Otherwise, they would desorb, and a sulphur-deficient MoS_2−x_ film would be formed. To maintain the stoichiometry of MoS_2_, the S/Mo ratio arriving at the substrate should be >2. To maximise the sticking coefficient of S in our process, we kept the sputter power as low as possible, at about 20 W, and maintained the working distance as high as possible, at up to 10–15 cm.

**Figure 6:**
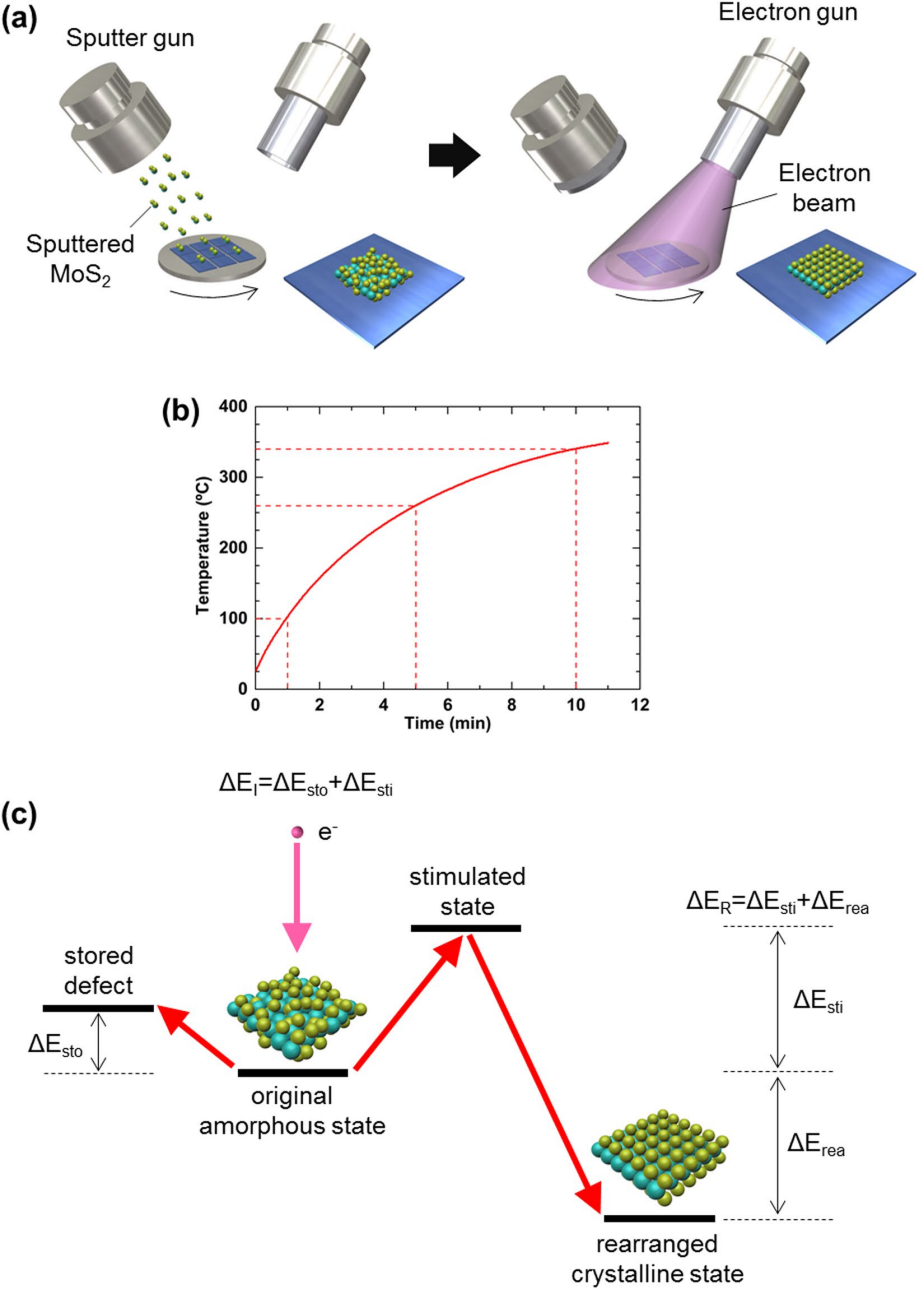
EBI process. (**a**) Schematic of the EBI process for the atomic rearrangement of MoS_2_ film from amorphous to a crystalline structure. (**b**) Substrate temperature profile under the EBI condition with an RF power of 300 W and DC power of 1 kV. (**c**) Mechanism for atomic rearrangement with EBI.

As shown in Figs 1 and 3, the Raman spectra and HR-TEM results confirmed that the as-deposited MoS_2_ film remained amorphous. However, we obtained a 2D layered structure for the MoS_2_ film after only electron irradiation with an energy of 1 kV for 1 min, as illustrated in Fig. 6(a). This is very surprising that such low-energy electrons with 1 kV encouraged atomic rearrangement for MoS_2_ crystallisation. No additional thermal heating process was applied to the substrate, and the temperature resulting from energetic electron bombardment of the substrate did not exceed 100 °C under the experimental condition of EBI with an energy of 1 kV for 1 min, as shown in Fig. 6(b). For the experimental results, the substrate temperature was measured by direct contact with a thermocouple. The substrate temperature exceeded 300 °C only after the EBI process for 10 min. These results confirm that atomic rearrangement for the crystallisation of a MoS_2_ film may occur at a much lower temperature than the conventional thermal crystallisation temperature with the EBI process at 1 kV for 1 min.

There have been several reports on crystallisation when using a focused high-energy electron beam (few tens of kilovolts to megavolts) on the localised area^25, 27, 28, 32^. Frantz *et al*. suggested a bond-breaking model for electron-irradiation-induced recrystallization in Si with an incident beam energy of 25 kV and claimed that the breaking of covalent bonds by electronic excitation and the re-bonding event itself can supply enough energy to the atoms surrounding the bond for structural rearrangement to occur^32^. Qin *et al*. reported a mechanism for electron irradiation-induced crystallisation of an amorphous Fe_85_B_15_ alloy system with an electron energy of 2 MeV^28^. They assumed that the electron-irradiated amorphous region is an open non-equilibrium system for which thermodynamic equilibrium is not applicable and that the external energy applied to an amorphous system can decrease the free energy and trigger an athermal transformation from an amorphous to crystalline state. By employing Qin *et al*.’s model for crystallisation with energy storage and dissipation in the electron-irradiated region, we can explain how an amorphous MoS_2_ structure can lower its internal energy to end up in a crystalline structure, as shown in Fig. 6(c). The incident electron beam (*∆E*_*n*_) irradiated on an amorphous MoS_2_ film is redistributed into two parts. One part is stored in the form of energy (*∆E*_*sto*_), which produces more unstable defects. The other part (*∆E*_*sti*_) is consumed to stimulate the atomic rearrangement of Mo and S atoms in the amorphous MoS_2_ structure. Because the stimulated states are thermodynamically unstable, those tend to lower their energies relative to the original amorphous state, which releases energy (*∆E*_*R*_) through atomic rearrangement into a crystalline MoS_2_ structure. In our experimental configurations for the crystallisation pathway of an amorphous MoS_2_ structure, the Raman and TEM results confirmed that an incident electron energy of 1 kV and irradiation time of 1 min were enough to stimulate this transformation despite the obviously low energy relative to previous works, as described above. Even though the electron energy of 1 kV seems to be too low to supply energy for bond-breaking and re-bonding in an amorphous MoS_2_ structure, it may be possible for MoS_2_ films with a thickness below 5 nm.

**Figure 7:**
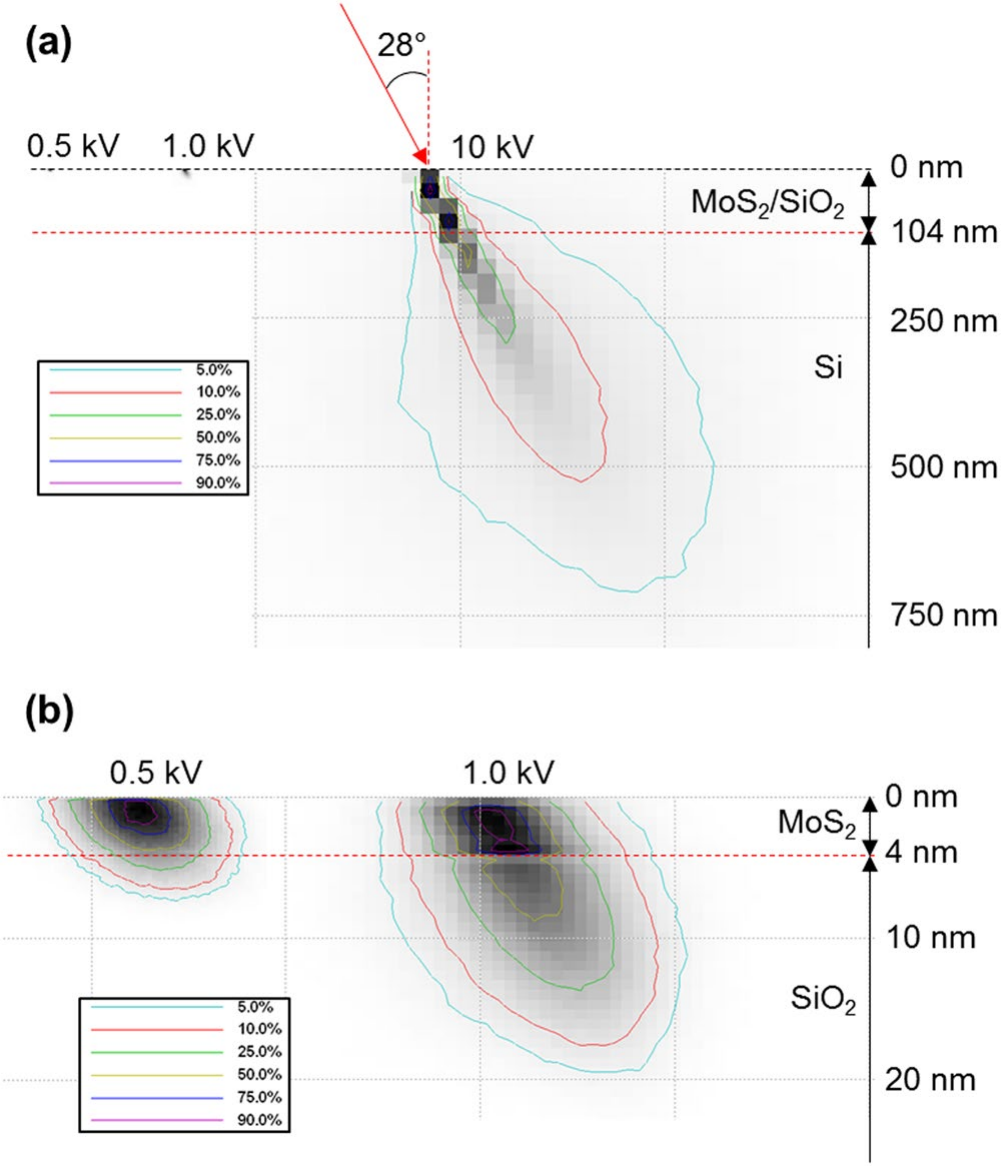
Energy absorption. (**a**) Cross-sectional view of the absorbed energy for the MoS_2_/SiO_2_/Si substrate according to incident electron energies of 0.5, 1.0, and 10 kV with an incident angle of 28° on the *x*–*z* plane. (**b**) Magnified image of the absorbed energy for the incident electron energies of 0.5 and 1.0 kV.

Figure 7 shows a CASINO simulation of the absorbed energy in a MoS_2_/SiO_2_/Si sample for different electron energies with an incident angle of 28° using our system^33^. The graph depicts the trajectory of electrons along the cross-section of a MoS_2_ film (4 nm) on a SiO_2_ (100 nm)/Si substrate. Figure 7(a) shows that the penetration depth increased with the incident electron energy. Most electrons with an energy of 0.5 kV did not exceed a depth of 7 nm and dissipated in the MoS_2_ layer, as shown by the magnified view of Fig. 7(b). At 1 kV, most electrons stopped moving within a depth of 20 nm; at 10 kV, a majority of the electrons were found in the Si substrate. In more detail, the MoS_2_ layer absorbed 50% and 25% of the energy at 0.5 and 1 kV, respectively, while less than 10% was absorbed at 10 kV. Therefore, the electrons with 0.5 kV energy seemed to be insufficient for transmitting all of the energy to a 4 nm thick MoS_2_ film, while those with excessive 10 kV energy almost permeated the thin MoS_2_ film and spent most of their energy in the Si substrate. For comparison with the CASINO simulation, we also derived the electron penetration depth by using the Potts range^34^:

1 $$x=\frac{0.1{E}^{1.5}}{\rho }$$

where *x* is the electron penetration depth (µm), *E* is the electron energy (kV), and *ρ* is the density (g/cm^3^). Theoretical calculations with Equation (1) revealed electron penetration depths of 6.9 and 19.8 nm with electron energies of 0.5 and 1.0 kV, respectively. These values are in good agreement with the simulation results given above. On the other hand, a penetration depth of 625.0 nm was observed for an electron energy of 10 kV.

Based on these results, the electron energy is a critical factor for the crystallisation pathway of amorphous MoS_2_ film during the EBI process. On the other hand, a long irradiation time and substrate heating during EBI are dominant parameters for damage to MoS_2_ films, such as sulphur loss^20^. Irradiation damage or modification of target atoms can be categorised in terms of the type of electron scattering, such as elastic and inelastic^26^. If incident electrons have sufficient energy, elastic scattering causes atomic displacement. For example, an electron energy of 100 kV is required to displace S atoms by elastic scattering for MoS_2_ films^35^. In the case of relatively low electron energy below a few kilovolts like in this study, inelastic scattering is the dominant type of modification for the crystal structure of target atoms^31, 32, 36–38^. For this type, it is almost impossible to displace atoms from a MoS_2_ bonding structure like with elastic scattering. Inelastic scattering is related to atomic loss, beam heating, local excitation, and bond breaking^25, 32^. Atomic loss via inelastic scattering can be caused by the Auger decay mechanism^26, 31^. In early studies on electron-induced crystallisation, beam heating through continuous electron irradiation was believed to be the main reason for the modification of the atomic structure. To date, however, results of experiments^37^ and simulations^32^ have led to the rejection of beam heating as a possible mechanism. Our experimental EBI configuration for MoS_2_ crystallisation and temperature measurement of the substrate, as shown in Fig. 6(a) and (b), respectively, also confirmed the rejection of beam heating because the substrate temperature increased to only 100 °C when an electron beam with 1 kV energy was irradiated for 1 min. Therefore, for the crystallisation pathway with our EBI process, bond breaking and local excitation are considered as possible mechanisms for the atomic rearrangement of the amorphous MoS_2_ film. As shown by the XPS spectra in Fig. 5, the Mo–Mo bond in the Mo 3d spectrum and S–S bond in the S 2p spectrum from the as-deposited film are weak and break easily. This may be due to the clustering during sputtering of the MoS_2_ target, which tended to form a stable Mo–S bond under the stimulated non-equilibrium conditions of the EBI process with 1 kV energy for 1 min. These bond breaking events in amorphous MoS_2_ film supply sufficient energy to the surrounding atoms to stimulate local excitation and provoke the atomic arrangement to lower their energies because the crystal-like bonding configurations are much more stable states. We deduced that the incident electron energy of the EBI process is a dominant factor for the crystallisation pathway of amorphous MoS_2_ film and that other parameters such as the irradiation time and substrate heating need to be optimised to minimise the damage and maximise the crystallinity of atomically thin MoS_2_ film. Even though a polycrystalline nature of the MoS_2_ film has been obtained under our experimental condition until now, it would be improved significantly by further optimisation of sputtering deposition and the EBI process and employment of specific substrate materials with an atomically flat surface such as hexagonal boron nitride (h-BN) and having a small lattice mismatch^39^ with MoS_2_ films such as hexagonal gallium nitride (h-GaN) and aluminium nitride (h-AlN).

## Conclusions

We presented experimental results showing the growth of crystalline MoS_2_ films after room-temperature sputtering with the EBI process. The amorphous as-deposited film was transformed into a crystalline MoS_2_ film after an EBI process for 1 min with an energy of 1 kV. Raman and HR-TEM results confirmed that 2D-layered MoS_2_ films with a hexagonal atomic arrangement formed on the SiO_2_/Si wafer despite the substrate temperature not exceeding 100 °C after electron bombardment. The FWHM values of the E^1^_2g_ and A_1g_ peaks in the Raman spectrum after the EBI process for 1 min were 19.3 and 11.5 cm^−1^, respectively. These are comparable to that of MoS_2_ film grown at a high temperature of 600 °C. The theoretical penetration depth of electrons with 1 kV energy for our experimental configuration was calculated to be 6.9 nm, which matches well with the CASINO simulation results. The thinness of the MoS_2_ film at less than 5 nm made crystallisation via the EBI process much easier, despite the relatively low electron energy of 1 kV. The synthetic method introduced in this work of sputtering and an EBI post-process can easily be applied to grow other TMDs such as MoSe_2_, WS_2_, and WSe_2_. It is a promising approach for achieving the low-cost and low-temperature applications based on flexible platforms, such as various sensors, photodetectors, and transistors.

## Methods

### Growth of MoS_2_ film

MoS_2_ films were grown by an RF magnetron sputtering system (Infovion Inc., Korea) with an electron beam source attached. A 50.8 mm diameter MoS_2_ target with 99% purity and Ar gas with 99.9999% purity were used for sputtering. Prior to MoS_2_ sputtering, SiO_2_/Si substrates were cleaned in an ultrasonic bath for 10 min in acetone, ethanol, and then isopropyl alcohol. The base pressure of the sputtering chamber was kept below 5 × 10^−7^ Torr, and the working pressure was maintained at 5 mTorr with an Ar flow of 10 sccm. MoS_2_ films were deposited at room temperature with an RF power of 20 W for 5 min. After the growth of the MoS_2_ films, EBI was performed for 1, 5, or 10 min. An electron beam with a 60 mm diameter was extracted from Ar plasma in the beam source and accelerated to the substrates. A highly dense induction coupled plasma was generated by RF power (300 W), and a collimated electron beam was formed with metal grids by DC power (1 kV).

### Characterisation of MoS_2_ film

The structural characterisation of the MoS_2_ films was performed by using HR-TEM for the cross-sectional view (Jeol, JEM-2100F (200 kV) and plan view (Jeol, JEM-4010 (400 kV)). A focused ion beam (FEI) was used to form a cross-sectional TEM sample of the MoS_2_ film after the EBI process for 1 min. We used a hole-filled carbon grid to prepare a plan-view TEM sample. In addition, Raman spectroscopy (WITec, alpha 300 S) with 532 nm laser and AFM (WITec, alpha 300 S) were used to check the lattice vibration and surface topography, respectively. Raman spectra were measured by using a × 100 objective lens and 1800 grooves/mm grating. The wavelength resolution of the spectrometer was 1.4 cm^−1^. The chemical state and stoichiometry of MoS_2_ films were characterised by XPS (Ulvac-PHI, PHI 5000 VersaProbe) with an Al K_α_ X-ray and pass energy of 23.5 eV for analysis.

### Simulation of Electron Penetration

A Monte Carlo simulation program (CASINO v2.48) was used to investigate the penetration behaviour of electrons in a MoS_2_ film on a SiO_2_/Si substrate. The incident angle and radius of the electron beam were set to 28° and 10 nm, respectively. A sufficient number of electrons (50,000 ea) was used for the simulation of absorbed energy, and their trajectories in the samples were displayed according to the beam energy. Backscattered electrons were not displayed in the results.
